# *Reticulamoeba* Is a Long-Branched Granofilosean (Cercozoa) That Is Missing from Sequence Databases

**DOI:** 10.1371/journal.pone.0049090

**Published:** 2012-12-04

**Authors:** David Bass, Akinori Yabuki, Sébastien Santini, Sarah Romac, Cédric Berney

**Affiliations:** 1 Department of Life Sciences, The Natural History Museum, London, United Kingdom; 2 Department of Zoology, University of Oxford, Oxford, United Kingdom; 3 Structural and Genomic Information Laboratory, CNRS, UMR 7256, Mediterranean Institute of Microbiology, Aix-Marseille University, Marseille, France; 4 CNRS, UMR 7144, Equipe Evolution du Plancton et Paleo-Oceans, Roscoff, France; Université Paris Sud, France

## Abstract

We sequenced the 18S ribosomal RNA gene of seven isolates of the enigmatic marine amoeboflagellate *Reticulamoeba* Grell, which resolved into four genetically distinct *Reticulamoeba* lineages, two of which correspond to *R. gemmipara* Grell and *R. minor* Grell, another with a relatively large cell body forming lacunae, and another that has similarities to both *R. minor* and *R. gemmipara* but with a greater propensity to form cell clusters. These lineages together form a long-branched clade that branches within the cercozoan class Granofilosea (phylum Cercozoa), showing phylogenetic affinities with the genus *Mesofila*. The basic morphology of *Reticulamoeba* is a roundish or ovoid cell with a more or less irregular outline. Long and branched reticulopodia radiate from the cell. The reticulopodia bear granules that are bidirectionally motile. There is also a biflagellate dispersal stage. *Reticulamoeba* is frequently observed in coastal marine environmental samples. PCR primers specific to the *Reticulamoeba* clade confirm that it is a frequent member of benthic marine microbial communities, and is also found in brackish water sediments and freshwater biofilm. However, so far it has not been found in large molecular datasets such as the nucleotide database in NCBI GenBank, metagenomic datasets in Camera, and the marine microbial eukaryote sampling and sequencing consortium BioMarKs, although closely related lineages can be found in some of these datasets using a highly targeted approach. Therefore, although such datasets are very powerful tools in microbial ecology, they may, for several methodological reasons, fail to detect ecologically and evolutionary key lineages.

## Introduction

The genus *Reticulamoeba* was created in 1994 by the distinguished protozoologist Karl Grell, in which study a single species, *R. gemmipara* was described [1]. This was followed in 1995 by a second species description, *R. minor* [2]. Both described species were isolated from the Mediterranean marine littoral zone, associated with diatoms, on which they feed. Both species are amoebo-flagellate; they have a stationary, more or less flattened amoeboid stage, of roundish to irregular outline and measuring c. 3–8 µm across. Thin reticulopodia radiate outwards from around the cell across the substrate, fusing at points to form networks that radiate out across the substrate. The area covered by the granular reticulopodia can be orders of magnitude greater than that occupied by the cell itself. Bidirectionally streaming granules (‘Körnchen’) can be seen on the reticulopodia. The reticulopodia themselves can move slowly, rearranging the size and shape of the network formed. When feeding, the reticulopodia penetrate diatom frustules rather than phagocytosing whole diatoms. Grell observed that networks from different individuals can fuse with each other, forming ‘feeding communities’, at least in *R. gemmipara*. He also describes a bi-flagellate stage, which is initially roundish in shape, becoming more irregular. These ‘swarmers’ or ‘zoospores’ have short anterior and long posterior flagella, and swim by active beating of the anterior flagellum, the posterior trailing behind. The flagellates can both swim and glide across a surface. They eventually settle, resorb their flagella and issue reticulopodia from around the cell, thereby transforming to the amoeboid stage. The main differences between *R. gemmipara* and *R. minor* are a) the flagellate and amoeboid stages of the latter are smaller, b) flagellate formation in *R. minor* occurs by fission of the amoeboid stage, resulting in two, four, or more zoospores, whereas in *R. gemmipara* zoospores are formed by unequal fission (budding) from the edge of the amoeboid cell.

The reticulate amoeba morphotype is generally very poorly known, and most studies concerning them fall into three main categories: 1) the original descriptions, usually without molecular data and in some cases ambiguous; 2) reports by other authors, often in passing or in a context where the prime focus is not the amoeba in question; and 3) more recent studies where a morphological description is complemented by the sequence of at least one phylogenetic marker gene and phylogenetic analysis. Since Grell’s work (category 1) there has been no definite re-recording of *Reticulamoeba*. The genus has been cited in passing a few times, e.g. [3]–[5], but never based on a robust identification (category 2). As of June 2012, five strains currently or previously referred to as *Reticulamoeba* are present in GenBank (JJP-2003 and COHH 9, 96, 98, 99). The first corresponds to *Filoreta marina* [6], and all the others are very closely related to it and therefore not *Reticulamoeba*. In this paper we show that *Reticulamoeba* is in fact a granofilosean cercozoan, and provide for the first time reliable 18S rDNA sequences for this genus, as well as describing morphological characteristics of novel lineages (category 3).

We used our new sequences to investigate the diversity and ecological distribution of *Reticulamoeba* further, by constructing and analyzing environmental SSU rDNA clone libraries, e.g. [7] using lineage-specific primers, by searching online sequence databases (e.g. NCBI GenBank nucleotide collection), and by mining 454 Sequencing datasets for sequences related to our cultured strains. The advent of high throughput, massively parallel sequencing technologies applied to environmental samples is currently revealing an even greater diversity of protist lineages than that indicated by ‘classical’ environmental cloning methods [8]–[11]. Strikingly, despite screening hundreds of millions of SSU rRNA gene sequences derived from samples that our cell isolation work suggested should be relatively rich in *Reticulamoeba*, we did not find any sequences matching known *Reticulamoeba* sequence types.

## Materials and Methods

### Sample Collection, Culture Isolation and Microscopy

Benthic samples for cell isolation and DNA extraction were taken from Port Swtan (Church Bay) Anglesey, Wales, UK (53°22′25″ N, 4°33′17″ W)), Walney Island, Cumbria, UK (54°03′04″ N, 3°11′18″ W), Thurlestone Beach, Devon (50°15′ N, 3°51′ W), and Chesapeake Bay, Queenstown, Maryland (38°59′ N, 76°10′ W). Other DNA samples were obtained from colleagues from the Colne Estuary [12], coastal sediment/rock scrapings from the eastern US seaboard between North Carolina and Washington DC, and recently formed biofilms in an experimental flume system in the River Lambourn, Berkshire. DNA and cDNA samples were also obtained from the BioMarKs consortium (of which DB and CB are members), collected as described in [9].

Samples for cell isolation were hydrated with dilutions of CCAP Artificial Sea Water Medium (ASW) and grown at room temperature without enrichment for a few days to a few weeks. Depending on the concentration of organisms, a 10–100 µl aliquot was then serially diluted across eight or twelve wells of 250 µl of ASW in a 96-well cell culture plate (Nunclon), with mixed marine diatoms as food source. The plate was then incubated at room temperature for a few days to a couple of weeks. Two or three rounds of serial dilution were carried out for each isolate, using only apparently pure strains to seed the final round.

**Figure 1:**
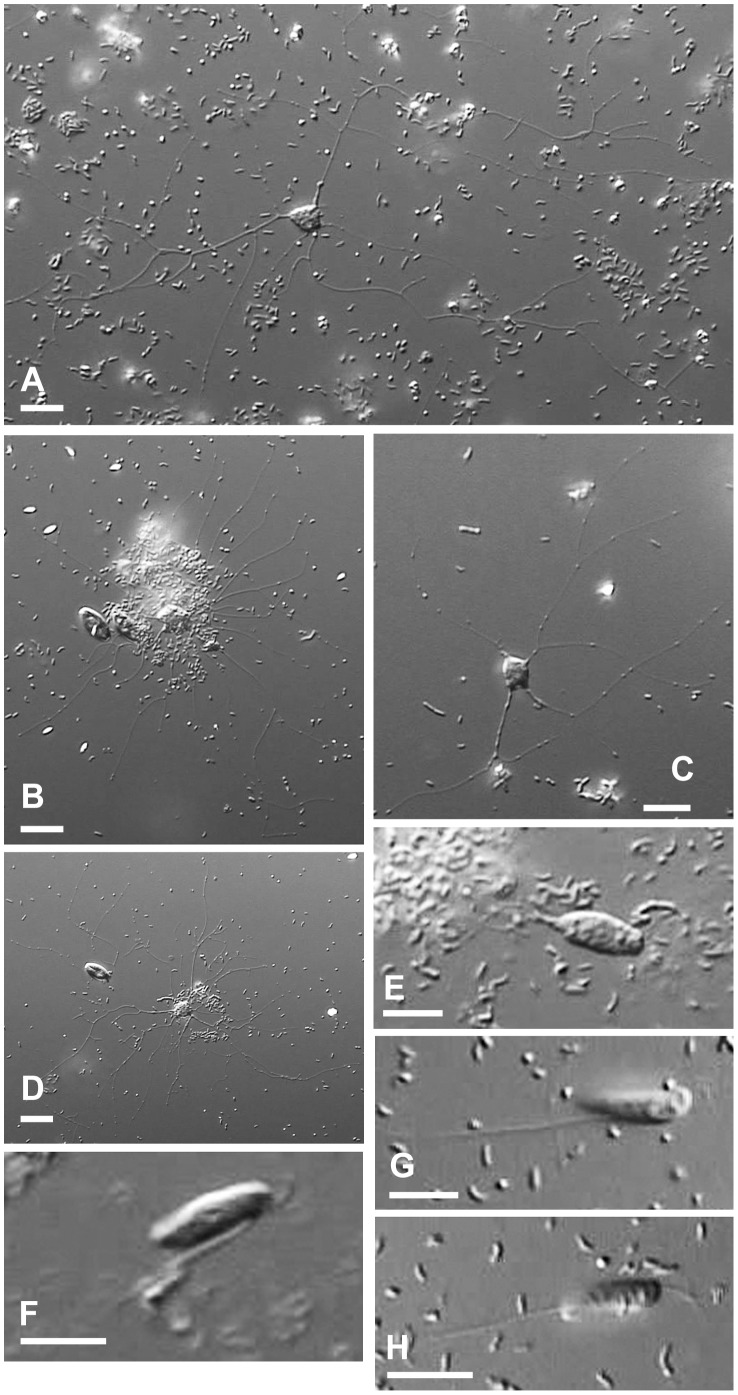
Different forms and growth stages of *Reticulamoeba minor* (Lineage 1; Isolates 1–3). **1A**–**1D**. Mature amoeboid cells with granular reticulopodia emerging from and radiating around cells. In **1B** and **1D** potential food sources (diatoms and bacteria) are associated with the *Reticulamoeba* cells. **1E**–**1H**. Swimming/gliding flagellate stages. **A**, **B**, **D**, **E** = Isolate 1; **C** = Isolate 3; Isolates 1–3 were phenotypically indistinguishable. Scale bars (A-D)  = 10 µm; (E-H)  = 5 µm.

**Figure 2:**
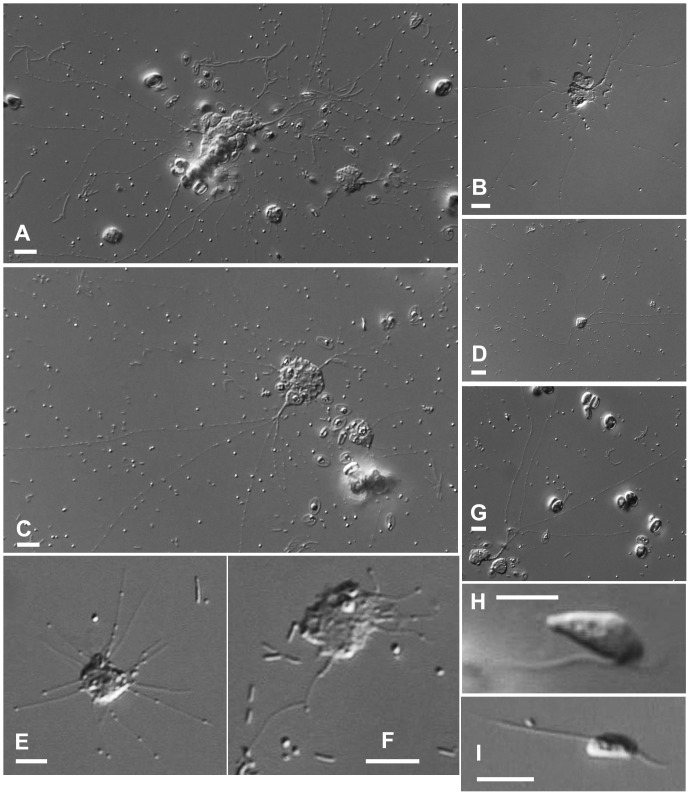
Different forms and growth stages of *Reticulamoeba gemmipara* (Lineage 4; Isolates 6 & 7). **2A**–**2C**. Mature amoeboid cells with forming ‘daughter’ cells. **2D**–**2G**. Earlier stage cells, including (**2E**, **2F**) cells formed within an hour of flagellate forms settling. **2H**–**2I**. Swimming/gliding flagellate stages. All images of Isolate 6, except D (Isolate 7). Scale bar  = 10 µm.

**Figure 3:**
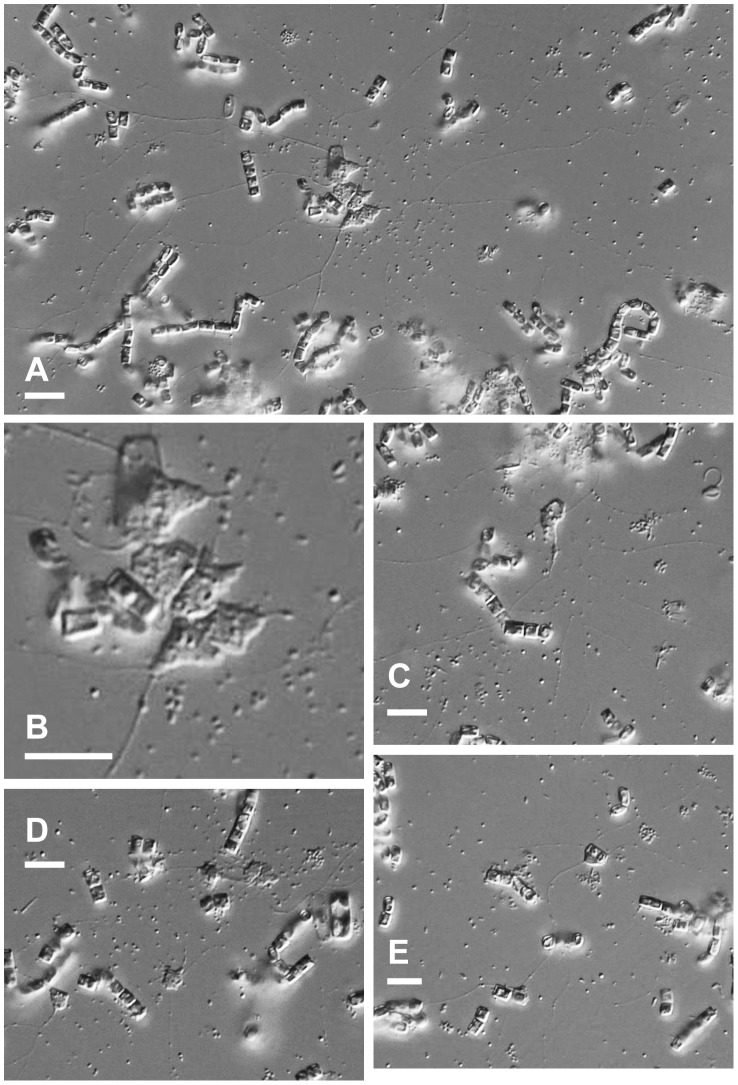
Amoeboid form of *Reticulamoeba* Isolate 4 (Lineage 2). Flagellate forms not shown but very similar to *R. minor* and *R. gemmipara*. The cells of this isolate were always strongly associated with diatoms, as in these photos. Scale bar  = 10 µm.

Live cultures were filmed and photographed using a Nikon Eclipse 80i microscope, with a x40 differential interference contrast water immersion lens (NA 0.6) and a Sony HDV 1080i Handycam®. Films were analysed on Final Cut Express HD 3.5.1, and digital images were exported and transferred to Adobe Photoshop for processing (Figures 1 to 3).

For DNA extraction, most of the culture medium was decanted off, and using a sterile scraper, cells were collected from the bottom of the culture dish, then concentrated by centrifugation at 24×g for 15 min at 5°C. Total DNA was extracted from the pellet following the Maximum Yield Protocol of the UltraClean Soil DNA Isolation Kit (MoBio Laboratories, CamBio, UK).

Ethics statement: No specific permission or permits were required for the described field studies. The sites were not privately owned or protected in any way and were fully open to public access. No endangered or protected species were involved in this study.

### Amplification and Sequencing of the SSU rDNA

PCR amplifications were done in a total volume of 30 µl with an amplification profile typically consisting of 35 cycles with 30 s. at 95°C, 30 s. at 60°C, and 90 s. at 72°C, followed by 5 min. at 72°C for the final extension. PCR products were run on 1.5% TAE agarose gels. Bands of the appropriate lengths were excised, and cleaned following the protocol of the QIAquick® Gel Extraction Kit (Qiagen). PCR amplicons were then cloned into StrataClone™ SoloPack® Competent Cells using the StrataClone™ PCR Cloning Kit (Stratagene). White colonies were screened using the primers M13for (5′-CGT TGT AAA ACG ACG GCC AGT-3′) and M13rev (5′-CAC AGG AAA CAG CTA TGA CCA-3′). Positive PCR products were cleaned using a polyethylene glycol (PEG) protocol: for 20 µl PCR reactions, 20µl of a 20% PEG/2.5 M NaCl mixture was added to each tube. The tubes were mixed by vortexing and incubated for 30 min at 37°C, then centrifuged at 3000 rpm for 30 min to pellet the PCR products. Supernatant was discarded by pulse-spinning the inverted tubes at 600 rpm. The pellet was then washed with ice-cold 75% ethanol, spun for ten minutes at 3000 rpm, again inverted and pulse-spun to remove the supernatant. The ethanol wash was repeated; the PCR pellet was re-suspended in de-ionised water, and stored at –20°C. Sequencing was performed with the ABI-PRISM Big Dye Terminator Cycle Sequencing Kit, and analysed with an ABI-377 DNA sequencer (Perkin-Elmer, Rotkreuz, Switzerland).

The first sequences were obtained from isolate 7 (*R. gemmipara* from Walney Island). Most of the usual combinations of universal or cercozoan-specific primers regularly used before, e.g. [13] didn’t lead to any good amplicons. Any strong band of the expected size proved to be from the diatoms on which the *Reticulamoeba* cells were feeding. We then focused on trying to amplify shorter fragments only, using a combination of cercozoan-specific and/or anti-diatom primers (Table S1). For three of the resulting PCR products, a faint band longer than the expected size was observed: n3NDf –1256R, s12aSf – sB2n, 1259F – sB2n. Direct sequencing of these bands proved impossible, so we cloned them and finally obtained the first real *Reticulamoeba* sequences. Two reverse primers specific for that isolate (V4r-d5a and V4r-d5b) were designed in the V4 region to amplify the missing first third of the gene with a nested PCR approach using forward primers sA1n and sA3n, respectively. Having sequenced the complete SSU rDNA of that first *Reticulamoeba* isolate made it possible to design an updated version of cercozoan-specific reverse primer 1256R, taking into account *Reticulamoeba*-specific substitutions (s1256R-d5), as well as a new granofilosean-specific forward primer (sA4-gran), to amplify the first two thirds of the SSU rDNA gene from all other isolates. This revealed the presence of four distinct SSU rDNA types in our isolates. Based on these four sequence types, two pairs of *Reticuloamoeba*-specific forward (V2f-d5 and C3f-d5) and reverse (V5r-d5a and V5r-d5b) primers were designed to construct *Reticulamoeba* clone libraries from environmental DNAs using a nested PCR approach. The resulting fragment (C3f-d5 to V5r-d5b) is about 800 bp. The two forward primers were also used to obtain the missing last third of the gene from one isolate of each identified species, again using a nested PCR approach, together with universal reverse primer sB1n and an updated version of primer sB2n (sB2-d5), respectively.

New sequences were deposited in GenBank with Accession numbers KC109661-KC109732.

### Construction of the Datasets and Phylogenetic Analyses

**Figure 4:**
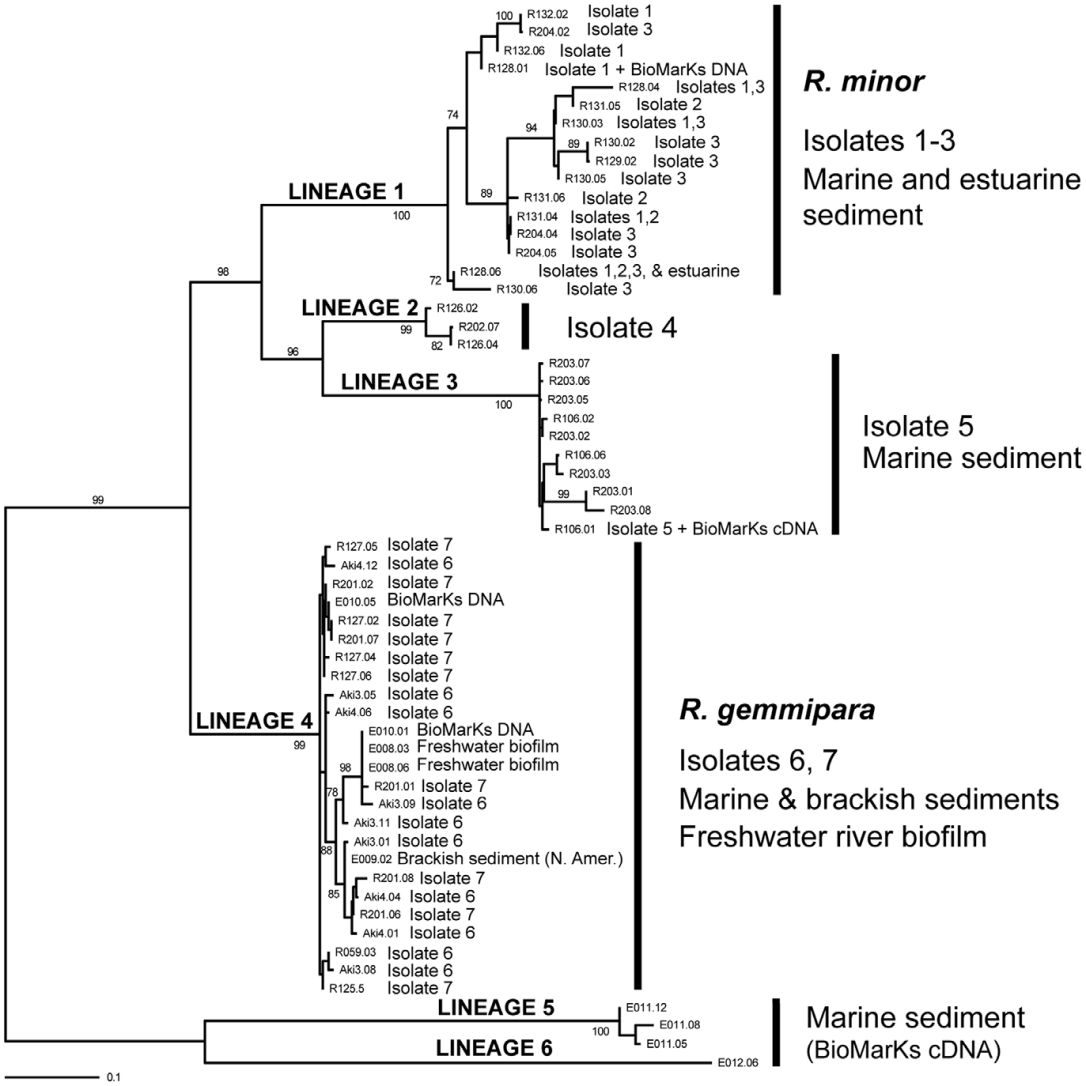
Maximum Likelihood (RAxML) SSU rDNA phylogeny of *Reticulamoeba* isolates 1–7 and the six main lineages identified in this study. 58 sequences, 1067 positions. ML bootstrap values shown when >70%.

**Figure 5:**
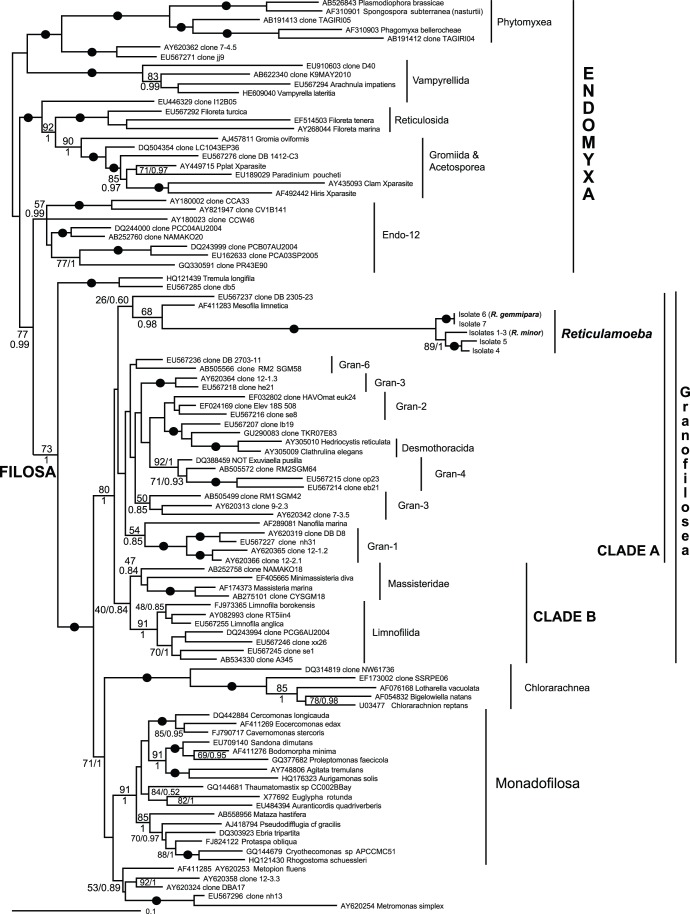
Bayesian SSU rDNA phylogeny of *Reticulamoeba* isolates (Lineages 1–4) and other Granofilosea in a cercozoan context. Support values shown when above the following thresholds: Bayesian posterior probabilities >75; ML bootstrap support >50%; or lower when of particular interest for the branching order within Granofilosea. Black filled circles indicate support of >95% bootstrap and 0.95 posterior probability. Gran- and Endo- clade designations refer to Bass et al. (2009).

BLAST searches [14] were performed using our new *Reticulamoeba* SSU rDNA sequences and revealed that exact matches were not present in the GenBank database. The closest sequences belonged to members of the phylum Cercozoa (Rhizaria). This was confirmed by preliminary trees including a wide range of eukaryotes, showing that in spite of their sequence divergence, our *Reticulamoeba* isolates clearly belong to the cercozoan subphylum Filosa. Two datasets were constructed for phylogenetic analyses. The first one, Figure 4 is restricted to *Reticulamoeba* sequences only so that the more variable regions of the gene could be included to illustrate the levels of inter- versus intra-specific sequence heterogeneity in that genus. It corresponds to a fragment of the SSU rDNA from forward primer C3f-d5 to reverse primer s1256R-d5 (1067 unambiguously aligned positions). This dataset includes all clone sequences we obtained from our *Reticulamoeba* isolates, plus the sequences obtained in our environmental libraries. The second (Figure 5; 1505 unambiguously aligned positions) includes the complete SSU rDNA sequences from five *Reticulamoeba* isolates, corresponding to four distinct SSU rDNA types, with two distinct isolates of type *R. gemmipara*. Sequences of representatives of all main cercozoan lineages were included, to determine the exact affiliation of *Reticulamoeba* within that phylum.

All phylogenetic analyses were performed using the GTR model of substitution [15], [16], taking into account a gamma-shaped distribution of the rates of substitution among variable sites, with eight rate categories. All necessary parameters were estimated from the datasets. For each dataset, a maximum likelihood (ML) tree [17] was determined with the program RaxML [18], using 250 inferences from distinct maximum parsimony starting trees. The reliability of internal branches was assessed with the bootstrap method [19]; 200 non-parametric bootstrap replicates with 10 inferences for each from distinct maximum parsimony starting trees (option –b –# 200–u 10). In addition, Bayesian analyses were performed with MrBayes version 3.1 [20], [21]. For each dataset, two runs of four simultaneous chains were run for 2,500,000 generations (heat parameters set to default), and trees were sampled every 100 generations. For each run 25,000 trees were sampled, 5,000 of which were discarded as the burn-in. Posterior probabilities of the branching pattern were estimated from the 40,000 remaining trees and mapped onto the maximum likelihood tree when present. In all cases, the posterior probability 50% majority-rule consensus tree was fully compatible with the corresponding ML tree. The dataset for Figure S1 was analysed using the RaxML BlackBox (v. 7.3.1) hosted on the Cipres Science Gateway (www.phylo.org/portal2/; [22]) only.

### Sequence Dataset Mining

The following datasets were blastn-searched for SSU rRNA gene sequences related to the *Reticulamoeba* genotypes as determined above: 1) NCBI GenBank nr/nt, 2) ‘All Metagenomic 454 Reads (N)’ in the CAMERA database (http://camera.calit2.net/; [23], [24]), 3) NCBI Environmental Sample Nucleotides (env_nt) via CAMERA, and 4) BioMarKs V4 SSU rDNA sequences generated using eukaryote-wide primers as described in [9]. For blastn searches against the NCBI and CAMERA databases, seed sequences were generated for each of the SSU-types recovered from the cultured strains by roughly dividing the longest SSU read into quarters and then generating three more fragments of similar size overlapping the boundaries of the original quarters. A fragment of c. 350 bp was also generated spanning the most variable V4 region and more conserved flanking regions with a strong signal for the *Reticulamoeba* clade.

## Results

### Morphology and SSU rDNA Phylogeny

We isolated seven strains of *Reticulamoeba*, and obtained the SSU rDNA sequence for each. Three of the isolates (Isolates 1–3; Figure 1) corresponded to Grell’s description of *R. minor*, and two (Isolates 6 & 7; Figure 2) to *R. gemmipara*. Isolate 4 (Figure 3) was intermediate between these two species in amoeboid cell morphology (although the mode of fission was not seen), and Isolate 5 (which died quickly in culture and is therefore not illustrated) had an amoeboid phase that itself was reticulate, i.e. several lacunae formed within the cell body, so that the cell was composed of a small network of cytoplasmic strands 1–3 µm wide; the overall size of the cell was larger than the other isolates. Isolates 1, 4, and 6 were from Church Bay samples; 2, 5, and 7 from Walney Island, and 3 from Thurlestone beach. Different isolates within a Lineage were morphologically and behaviourally indistinguishable; the choice of images in Figs. 1–3 is based on their suitability for illustration.

Because all isolates had relatively high levels of intra-genomic SSU rDNA sequence diversity, the PCR products were cloned and the resulting sequences analysed phylogenetically. Figure 4 shows that the sequences recovered from Isolates 1–3 form a clade (Lineage 1 ( =  *R.minor*)) in which there is no apparent phylogenetic distinction between the three isolates. Cloned sequences from Isolates 4 and 5 (sister Lineages 2 and 3, which together are sister to Lineage 1) each also form clades, though will lower internal sequence diversity than Lineage 1. Isolates 6 and 7 (Lineage 4 ( =  *R. gemmipara*)) showed a similar molecular diversity pattern to Lineages 2 and 3. It is possible that the apparently high intra-genomic SSU diversity and mixed genotypes across Isolates 1–3 could be explained by there being more than one *Reticulamoeba* lineage in the sequenced isolates. However, we think this unlikely as this pattern only relates to these three lineages (there is no mixing of these sequence types with any other lineages or between any of the other lineages), and distinctive sequence signatures in particularly variable regions along the SSU of isolates 1–3 show a mosaic distribution across the three isolates suggesting a partly reticulate evolutionary history of these lineages. The simplest explanation is that these three isolates are a single evolutionary unit and species: *R. minor*.

The morphology of *Reticulamoeba* strongly suggests a cercozoan affinity, specifically with Granofilosea, which share its granular filopodia-like amoebo-flagellate characters, although the granules on other Granofilosea move very much less than in *Reticulamoeba*, or not at all [6]. We were surprised therefore when DNA extracted from *Reticulamoeba* did not amplify with any of the several primer sets known to amplify most filosan Cercozoa (see Methods). The difficulty of obtaining SSU rDNA sequences from *Reticulamoeba*, requiring several new primer sets and ‘walking’ along the SSU rRNA gene was explained by the highly divergent nature of the sequences, represented by the long branch leading to the *Reticulamoeba* radiation in Figure 5. This tree does, however, confirm that *Reticulamoeba* branches within Granofilosea, most consistently as sister to the freshwater genus *Mesofila*. The affinity is concordant with the morphology and lifestyle of *Mesofila*, from which it differs in three main respects: 1) the filopodia of *Mesofila* are not, or are much less, anastomosing than those of *Reticulamoeba*, 2) the filopodial granules in *Mesofila* are stationary, and 3) habitat. However, like *Reticulamoeba*, *Mesofila* readily forms gliding/swimming flagellate forms, to a much more noticeable extent than most other naked Granofilosea.

### SSU rDNA of Reticulamoeba: Sequence Divergence and Intra-specific Heterogeneity

The *Reticulamoeba* SSU rDNA sequences obtained in this study proved unusual in many respects. Even though they possess most typical cercozoan- and filosan-specific sequence signatures, they appear to be very divergent compared to the sequences of other Filosa, with very many specific substitutions distributed across the whole length of the gene. Secondly, as suggested by the size of all amplicons, they are indeed longer than the average size of the gene in most eukaryotes, with specific insertions in many variable regions. Finally, there proved to be a surprisingly high level of intra-genomic heterogeneity between different copies of the SSU rDNA gene within all sequenced *Reticulamoeba* isolates. This heterogeneity is significantly higher than any we are aware of in other amoeboid organisms, including both size and primary sequence variation. Several different sequence patterns can be observed in all variable regions of the gene, and the different clone sequences we obtained for each isolate appear to correspond to random combinations of these patterns. This is the reason why cloning proved to be necessary for all amplicons we sequenced.

Importantly, the observed sequence heterogeneity within isolates (and between isolates sharing the same morphology) is limited to the most variable regions of the SSU rDNA. Even though surprisingly high, it remains significantly lower than the observed sequence heterogeneity between isolates exhibiting different morphologies. Therefore we could readily assign each of our isolates to a well-defined SSU rDNA type, and these correlated perfectly with a morphological type, and assumedly by extension to different species. By contrast with intra-specific heterogeneity, inter-specific differences extend to less variable regions of the gene, but are very conserved within species. This is illustrated by both tree figures. In figure 4, we can see that the various clone sequences from isolates of a same morphological type are intertwined and exhibit the same levels of sequence heterogeneity. In figure 5, we can see that once the most variable regions have been excluded for phylogenetic analyses including members of all main cercozoan lineages, sequences from different isolates of the same species are otherwise identical, while significant differences can be observed between isolates corresponding to distinct morphological types/species.

### Diversity and Ecology of Reticulamoeba

Although *Reticulamoeba* cells can be difficult to see in crude environmental cultures, we found them frequently when screening littoral benthic, particularly sandy or silty samples. Therefore we hypothesized that this genus is much more abundant than suggested by its minimal (and entirely specimen-derived: see below) representation in Genbank. To investigate this further, we designed *Reticulamoeba*-specific primers (see Methods) to screen environmental DNA extractions (each representing c. 0.5 - 1 g sediment) from eleven samples taken along an estuarine gradient, six newly-formed freshwater (river) biofilm samples, nine coastal sediment and rock scrapings from various sites on the US east coast between North Carolina and Washington DC, and BioMarKs coastal (offshore) sediment samples (nine DNA and nine cDNA, paired from the same set of sites) from Roscoff (France), Oslo (Norway), Barcelona (Spain), Varna (Bulgaria), and Naples (Italy). Of the estuarine samples, sample 6 (a midpoint estuary site; [12]) gave a positive PCR result, as did two of the river biofilm samples, one Maryland coastal sediment, and one BioMarKs sediment DNA sample. In contrast seven out of nine BioMarKs cDNA samples amplified. The branching position of the cloned sequences is shown on Figure 4. The library sequences exclusively grouped with the *Reticulamoeba* sequences derived from cultures, including two novel sister lineages (5 and 6; Figure 4) recovered only from the BioMarKs cDNA samples, which are sister to the cultured and other environmental sequences (as confirmed by Figure S1.). Lineage 6 had a shorter amplicon by c. 40 bp than lineage 5. Interestingly, we detected lineage 4 twice independently in freshwater river biofilm samples. This is intriguing since *Reticulamoeba* has never been seen or otherwise recorded from freshwater (as distinct from brackish water).

### Next Generation Sequencing Database Mining

Our environmental SSU clone libraries suggested that *Reticulamoeba* is more abundant and diverse than existing sequence data implied. To investigate whether this was reflected by its representation in massively high throughput next generation sequencing (NGS: 454, Illumina) datasets, we used full and partial SSU rDNA sequence ‘seeds’ to look for representatives/relatives of all six lineages in three main types of dataset: NCBI GenBank nucleotide database, 454 amplicon libraries generating using eukaryote-wide SSU primers, and NGS and Sanger-sequenced metagenomic, shotgun libraries from a range of marine and non-marine habitats, as hosted by CAMERA (see Methods).

In NCBI nr/nt the only blastn matches that were >95% similar to sequences in the database were in relatively conserved regions of the SSU rRNA gene, which are therefore taxonomically uninformative. However, even most of these could be rejected as one of the six *Reticulamoeba* lineages shown in Figure 4 because they lacked sequence signatures shared by all six lineages. When the region used as blast query was restricted to the most variable part of the V4 region (a 240-bp fragment, corresponding to positions 640 to 880 in the *Mesofila limnetica* (previously known as ‘*Dimorpha*-like’) SSU sequence AF411283) and the discontinuous megablast option applied the result was the same as with blastn: short matches (c. 40 bp only) to non-cercozoans with no more than 95% similarity. The NCBI env_nt dataset (∼20 M sequences) was interrogated via CAMERA and similarly only showed high identity matches in conserved regions.

**Figure 6:**
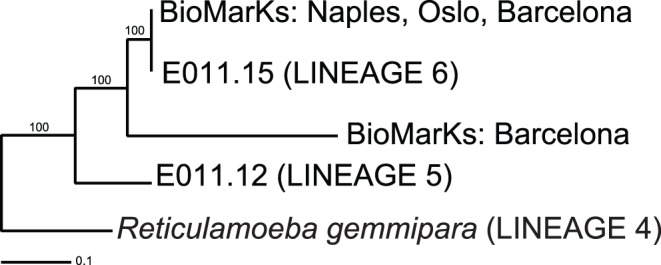
Maximum Likelihood (RAxML) SSU rDNA tree including sequences from BioMarKs. The BioMarKs (V4 region) sequences were generated using eukaryote-wide primers, and are labelled ‘BioMarKs: …’. The two such lineages shown were the only sequences in the whole of the BioMarKs data that were related to *Reticulamoeba*. 738 positions used for analysis.

The BioMarKs 454 sequenced SSU V4 data (c. 1.5 M sequences from coastal marine sites around Europe: Blanes, Gijon, Naples, Oslo, Roscoff, Varna; further details in Logares et al. (2012)) also contained no sequences in variable regions that were >90% similar to lineages 1 to 4. A useful *Reticulamoeba* clade-specific sequence signature is situated in the V4 region, at the 3′ end of the generally most variable stretch (at positions 806 to 809 in *Mesofila limnetica*. Here all six *Reticulamoeba* lineages have a unique CACA motif (*Mesofila* has AATA). The only BioMarKs V4 sequences containing the CACA signature are shown in Figure 6, which shows that in this entire very large dataset the only direct matches were to lineage 6. These sequences were derived from cDNA samples from benthic sediment from the Naples and Oslo sites only. Lineage 5 was not detected in these eukaryote-wide data. A new lineage was found, ‘D2IMK’ in the Barcelona samples, although there is a possibility that this is chimeric. The next closest blastn returns from the BioMarKs database included some novel granofilosean sequences, one very divergent one 83% similar to *Mesofila*, and others 93% similar to op32 (novel clade Gran-4; Bass et al. 2009) and 96% identity to eb6 (novel clade Gran-1).

The CAMERA All Metagenomic 454 Reads (227.3 M sequences) blastn search also returned only high identity matches in conserved regions of the SSU. Only returned sequences with >95% identity to the query sequences were investigated further. These included matches to other Cercozoa including the granofilosean sequence se8 (novel clade Gran-2; [6]) and the endomyxan sm5 (novel clade Endo-5).

## Discussion

We have frequently seen in crude environmental cultures and occasionally isolated *Reticulamoeba*-like amoeboflagellates that resisted amplification with primer sets that easily amplify most Cercozoa and, particularly Granofilosea. Therefore the difficulty of PCR-amplifying those strains that differed from other granofilosea in having bi-directionally streaming granules on their reticulopodia was a puzzle that was only resolved with intensive PCR attempts using a variety of primers and targeting short amplicons, and eventually largely sequencing by ‘walking’ along the SSU rDNA molecule. This resulting in associating a SSU sequence with Grell’s hitherto elusive *Reticulamoeba*, and showing that it groups robustly within Granofilosea (although the intra-granofilosean relationship requires confirmation with other genes as the SSU branch is very long and therefore likely prone to phylogenetic long branch attraction effects).

Our observations by microscopy and using lineage-specific primers strongly indicate that *Reticulamoeba* lineages are much more densely and widely distributed than currently available databases suggest. This is almost certainly because the SSU rDNA of *Reticulamoeba* is relatively difficult to amplify and is therefore biased against by more general PCR primers, for example those designed to detect a broad eukaryotic diversity. A robust confirmation of this is that none of the four unambiguously identified *Reticulamoeba* lineages (1 to 4) was recovered from the V4 BioMarKs data generated with eukaryote-wide primers, even though these have been shown to be broadly inclusive, e.g. [11]. However, one novel lineage (5) was detected in the general eukaryote V4 reads from two BioMarKs samples. By contrast, lineage-specific PCR probing of the same nucleic acid samples revealed five of the six lineages, the sixth being newly found by this specific PCR approach.

It is striking that *Reticulamoeba* lineages were detected in the BioMarKs samples with a strong bias towards the RNA-derived (cDNA) samples. All sequence types in Figure 6 (from the eukaryote-wide dataset) are RNA-derived, and the lineage-specific PCR was far more successful on cDNA samples compared to the twinned (same site and sampling point) DNA sample. Lineages 5 and 6 were only detected in BioMarKs cDNA samples. Lineage 3 was recovered from a single (now dead) isolate and otherwise only from lineage-specific probing of BioMarKs cDNA samples. The relative ease of amplifying from cDNA in preference to equivalent DNA has been noted before, even from culture isolates, e.g. [25], and it is well established that cDNA environmental diversity libraries often reveal a different subset of microbial communities than otherwise similar ones from DNA [26], [27] (i.e. some lineages appear to be more readily detected from a DNA template, and others from cDNA). cDNA synthesis removes introns, which complicates sequencing of some SSU rDNA, although this does not explain why the cDNA samples we amplified worked better than the corresponding DNA samples; we know from the DNA-derived sequences that there are no introns in the fragment of *Reticulamoeba* SSU that we amplified. However, our results emphasise that an RNA sample basis may be the only reliable way of detecting some lineages, and that even with very large sequencing efforts, primers with broad phylogenetic range can fail to detect important (and very interesting) elements of protistan community diversity.

Metagenomic (hereafter for simplicity used to refer to both true metagenomes, i.e. shotgun-sequenced genomic DNA without a PCR amplification step and shotgun sequenced RNA-derived metatranscriptomes) sequence libraries can theoretically better represent the composition of the communities from which they were constructed because they avoid PCR and its attendance biases [10]. Next generation sequencing technologies now offer a depth of sequencing that might offset the likelihood of extreme taxonomic undersampling because of the size and complexity of the whole genomes comprising microbial communities. However, none of the fragments of any of the six *Reticulamoeba* lineages, nor ‘full length’ SSU rDNA sequences matched any metagenomic sequence in the largest, most comprehensive metadataset hosted by CAMERA. This could be partly explained by the fact that, although it has a swimming flagellate stage, *Reticulamoeba* is far more obviously benthic than planktonic, and the marine datasets in CAMERA are very strongly planktonic. To gauge the sensitivity of this method of recovering SSU tags from metagenomic datasets we created an equivalent set of V4 blastn seeds from the three sequences of *Solenicola setigera* [28] from NCBI. *Solenicola* is a member of MAST-3 [9], which is the most highly represented of the planktonic marine stramenopile groups in the BioMarKs eukaryote-wide data. This suggests that it should be among the easiest groups to detect in similar marine metagenomic datasets. Indeed, we recovered many identical and highly similar V4 reads (>95% sequence identity across highly variable regions) from at least 36 individual metagenomic samples in CAMERA, by blastn-searching the three *Solenicola* V4s against the All Metagenomic 454 Reads. This shows (as do other studies such as Not et al. 2009) that it is a reasonable proposition to search for SSU tags in metagenomic datasets such as those hosted by CAMERA, and that *Reticulamoeba* is not represented because the samples do not cover its main habitat and/or it is insufficiently abundant in the samples to be detected in this way.

It remains a striking fact, therefore, that although there is good evidence for *Reticulamoeba* being a frequent and diverse element of marine benthic (at least) protist communities, no direct evidence can be found for it in any existing sequence dataset, even though these have been constructed using a diverse range of techniques (well representing all that are currently available) and harnessing the power of massively parallel next generation sequencing technologies. Culture-based investigations have recently revealed other elusive, long-branched Cercozoa (e.g. *Sainouron*, *Helkesimastix*, *Cholamonas*, *Guttulinopsis*; [29]–[31]) that require intensive and case-specific attention to yield genetic data that can be used to detect their presence in nature. How many more such lineages are there, and how diverse and abundant are they relative to those more easily detected by environmental screening techniques?

## Supporting Information

Figure S1 **Maximum Likelihood (RAxML) SSU rDNA phylogeny of** ***Reticulamoeba*** **clade within Cercozoa.** 37 sequences, 1656 positions. Showing the relative positions of *Reticulamoeba* isolates and novel environmental sequences from BioMarKs data.
(TIF)

Table S1 **Primers used in this study.**
(DOC)
