# Supplementary figures and images for: Reticulamoeba Is a Long-Branched Granofilosean (Cercozoa) That Is Missing from Sequence Databases
Source: PLoS One. 2012 Dec 4;7(12):e49090. doi: 10.1371/journal.pone.0049090 (PMC3514243; doi:10.1371/journal.pone.0049090)

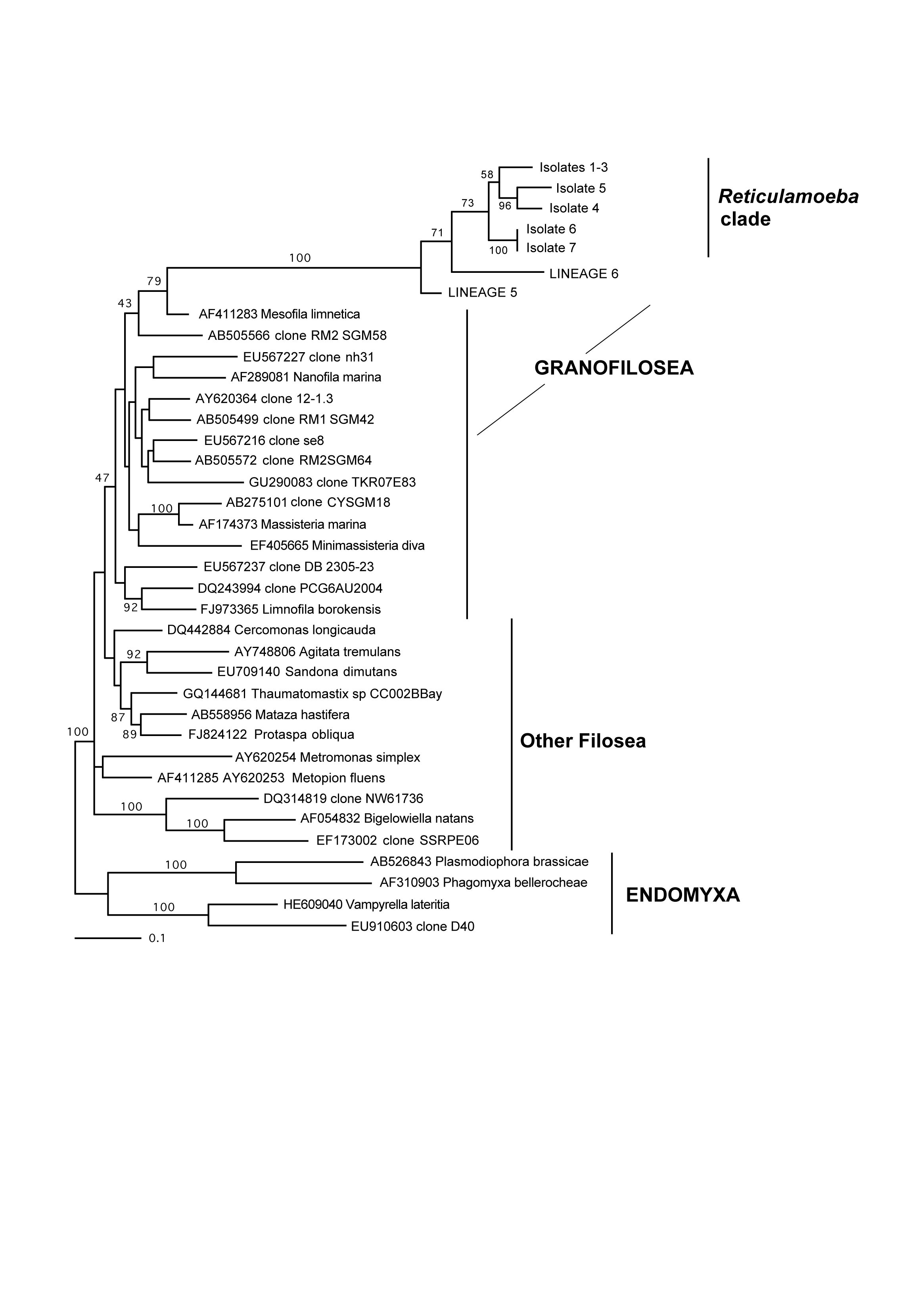

Supplement: Figure S1 — Maximum Likelihood (RAxML) SSU rDNA phylogeny of Reticulamoeba clade within Cercozoa. 37 sequences, 1656 positions. Showing the relative positions of Reticulamoeba isolates and novel environmental sequences from BioMarKs data. (TIF) [file pone.0049090.s001.tif]
