# Supplementary material for: Reticulamoeba Is a Long-Branched Granofilosean (Cercozoa) That Is Missing from Sequence Databases
Source: PLoS One. 2012 Dec 4;7(12):e49090. doi: 10.1371/journal.pone.0049090 (PMC3514243; doi:10.1371/journal.pone.0049090)
Supplement: Table S1 — Primers used in this study. (DOC) [file pone.0049090.s002.doc]

**Table S1. Primers used in this study.**

| primer name | direction | sequence | position * | specificity |
| --- | --- | --- | --- | --- |
|  |  |  |  |  |
| **sA1n** | forward | ACC TGG TTG ATC CTG CCA GT | 2-20 | universal (eukaryotes) |
| **sA3n** | forward | GCC AGT WGT SAT ATG CTT GTC THA | 15-38 | universal (eukaryotes) |
| **sA4-gra** | forward | CNG TGA AAC WGC AGA TGG | 79-96 | Granofilosea incl. *Reticulamoeba* spp. |
| **V2f-d5** | forward | GGA TAG CCG TAC TAA TTG TGG | 142-162 | *Reticulamoeba* spp. |
| **C3f-d5** | forward | GAC ATC TGA GGT GAT AAC GAA | 344-364 | *Reticulamoeba* spp. |
| **n3NDf** | forward | GAG GGM AAG YCT GGT GCC AGC AGC | 556-580 | universal (eukaryotes) |
| **V4r-d5b** | reverse | GGA TGA CAA TGT TTG CGG TGA | 740-760 | *Reticulamoeba* *gemmipara* |
| **V4r-d5a** | reverse | CTC GGA TTC CTG AAA CCA ATG | 850-870 | *Reticulamoeba* spp. |
| **s12aSf** | forward | CGA AGA CGA TCA GAT ACC GTC | 1007-1027 | anti-heterokonts |
| **V5r-d5b** | reverse | GTC AAC GCT CGC TGA TCC CTG | 1055-1075 | *Reticulamoeba* spp. |
| **V5r-d5a** | reverse | GGT GCC AAC GAG GTC GTT TCA | 1075-1095 | *Reticulamoeba* spp. |
| **1259F** | forward | GGT CCR GAC AYA GTR AGG ATT GAC AGA TTG AAG | 1229-1261 | most Cercozoa |
| **s1256R-d5** | reverse | CCA YMR AAT CAA GAA AGA DCT TCA | 1257-1280 | Cercozoa incl. *Reticulamoeba* spp. |
| **1256R** | reverse | GCA CCA CCA CCC AYA GAA TCA AGA AAG AWC TTC | 1258-1290 | most Cercozoa |
| **sB2-d5** | reverse | CCT TGT TAC GAC TTT TGC | 1774-1791 | *Reticulamoeba* spp. + some eukaryotes |
| **sB2n** | reverse | CCT ACG GHD ACC TTG TTA CGA CTT CTC | 1775-1801 | anti-heterokonts, -opisthokonts, -Filosa |
| **sB1n** | reverse | GAT CCH TCY GCA GGT TCA CCT ACG | 1796-1819 | universal (eukaryotes) |

* using the sequence of *Mesofila limnetica* (GenBank accession number AF411283) as a reference
